# The Density Profile of a Neutron Star

**DOI:** 10.3390/e27020194

**Published:** 2025-02-13

**Authors:** Allan D. Woodbury

**Affiliations:** Department of Civil Engineering, University of Manitoba, Winnipeg, MB R3T 2N2, Canada; allan.woodbury@umanitoba.ca

**Keywords:** neutron stars, density profile, inverse solution, minimum relative entropy

## Abstract

The problem posed in this study is to determine the density distribution within an ideal spherically symmetric neutron star based on only two constraints: the volumetrically averaged density and a moment of inertia factor, *f*. In order to deal with the above, it is recognized that space within these objects is heavily curved, and thus lengths, densities, and the moment of inertia have to be adjusted for relativistic effects. For the first time, the minimum relative entropy methodology (MRE) is used to find the expected value of a series of effective densities within a neutron star. In numerical experiments, we use the data from the star PSR J0737-3039A, which has a mass of $2.6\times 10^{30}$ kg and a radius of 13.75 km. Here, the factor *f* is based on a range of values of moments of inertia (MOI): 1.30–1.63 $\times 10^{45}$ g cm^2^. For f=0.324, at no time do densities cross over $1\times 10^{15}$ gm/cc. For the most part, densities > $6\times 10^{14}$ gm/cc are shown at radial dimensions of less than about 4 km. When f=0.258, densities closer to the core are pushed higher, as one might expect, and peak at slightly over $4\times 10^{15}$ gm/cc. If recent values of MOI are more appropriate at $1.15\times 10^{45}$ g cm^2^, this then suggests core densities greater than $4\times 10^{15}$ gm/cc. These various density models lead to quantitative statements about qualitative interpretations, and as time goes on, any internal density models should satisfy the two constraints posed. Also, since the model presented here is probabilistic, it can be established that density at a certain depth is constrained within a certain confidence limit. The expected values of densities for PSR J0737-3039A are in reasonable agreement with current conceptual neutron star models but are highly sensitive to assumed MOI values. It is emphasized that the probabilities and the mean values of density obtained are conditional on the imposed moments, namely, M and *f*, and also the radius R.

## 1. Introduction

**Figure 1:**
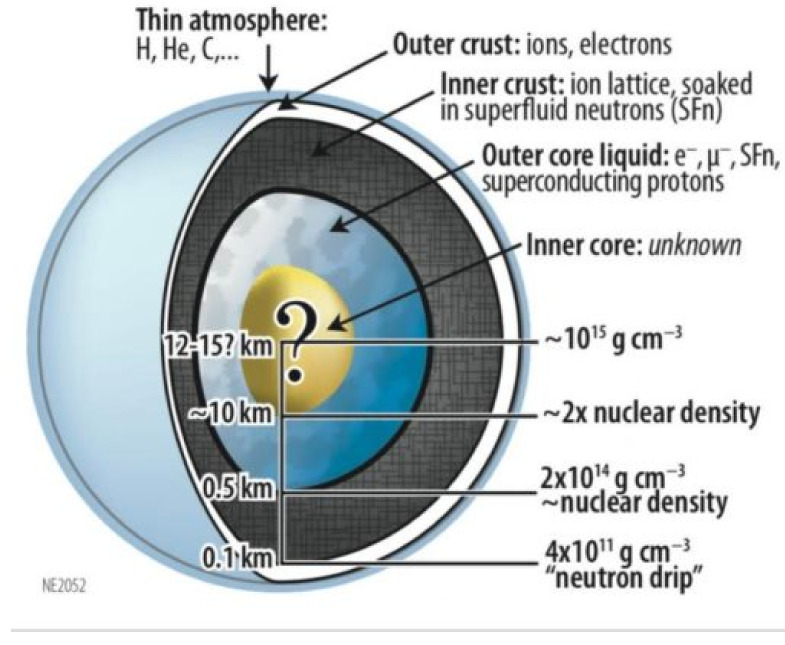
Conceptual model of a neutron star and its interior. Credit NASA/B. Link, https://heasarc.gsfc.nasa.gov.

The general consensus at this point is that the interior distribution of mass within neutron stars is unknown and highly speculative [1]. Figure 1 shows such a conceptual model of a neutron star (Source: NASA). It is unlikely that astronomical science will ever be able to obtain any direct data regarding the density of neutron stars using current or future astrophysical observations. However, these types of poorly posed problems can be attacked with information-based methods [2].

The methodology contained herein does not rely on solutions to the classic TOV equation [3], which relates internal matter and pressure to curvature in space. Instead, it is the goal of this work to estimate the internal density distribution of a neutron star using observations from distant observers and inference-based methods.

To approach this problem, we postulate an ideal case of a static, spherically symmetric neutron star of a known coordinate radius R and mass M (as measured by a distant observer). In this case, ρ=ρ(r) only. The moment of inertia factor *f* is also known or can be estimated. In dealing with this problem, it is noted that the space inside a neutron star is curved and, therefore, has a larger *relativistic* radius and volume than its Euclidean counterpart.

Consider a spherical object of uniform mass–density ρ and radius R with a gravitational potential Φ given by a solution to Poisson’s equation:

(1) $$\Phi \left(r\right)=\frac{2}{3}\pi G\rho \left[r^{2}-3R^{2}\right]$$

where G is the gravitational constant, *r* is the radial coordinate from the center of mass, and r<R. If $\Phi /c^{2}<<1$ (*c* is the speed of light), then relativistic effects need not be considered and the metric tensor can be expanded in terms of the gravitational potential. In the developments below, we find that for the stars considered, $\Phi /c^{2}<1$, thus requiring correction to the fundamental metric. To start, note the familiar metric where r,θ,ψ are the usual radial coordinates:

(2) $$ds^{2}=-c^{2}e^{\nu }dt^{2}+e^{\lambda }dr^{2}+r^{2}\sin^{2}\theta d\psi^{2}+r^{2}d\theta^{2}$$

In order to determine the constants λ and ν, one must solve a system of equations that relate the energy momentum tensor T to Einstein’s tensor G. Considering a perfect fluid assumption, the stress–energy tensor is diagonal with $T_{00}=\rho c^{2}$, zero off-diagonals, and diagonals 1 to 3 equal to −P. To proceed further, note the classic TOV derivation in which Einstein’s field equations are solved. These results are well known [3].

(3) $$\frac{8\pi G}{c^{4}}T=G$$

After some manipulation and only considering the $G_{00}$ component

(4) $$\frac{8\pi G}{c^{4}}\rho c^{2}e^{\nu }=\frac{e^{\nu }}{r^{2}}\left(1-\frac{d}{dr}\left[re^{-\lambda }\right]\right)$$

and solving for $e^{-\lambda }$:

(5) $$e^{-\lambda }=1-\frac{2Gm(r)}{rc^{2}}$$

where m(r) is the mass contained inside a radius *r*, as felt by a distant observer and is defined as

(6) $$m\left(r\right)=\int_{0}^{r}4\pi \rho \eta^{2}d\eta$$

Since in our case we are only interested in static conditions in the radial direction, the metric becomes

(7) $$ds^{2}={\left[1-\frac{2Gm(r)}{c^{2}r}\right]}^{-1}dr^{2}$$

Note that at this point, while a perfect fluid assumption (constant density, etc.) has been made, there is really no impediment to hypothesize that a stellar object comprises a series of layers (shells). In each layer, the density could be assumed constant, thus comprising a system of layered heterogeneity. It is worth noting that Schwarzchild’s constraint of a completely homogeneous sphere circumvented the implicit non-linearities in TOV and made mass calculations tractable. Other forms of the metric are, for example [4]:

(8) $$ds^{2}=\frac{dr^{2}}{\left(1-\alpha^{2}r^{2}\right)}$$

where α is defined as

(9) $$\alpha =\sqrt{\frac{S}{R^{3}}}$$

and

(10) $$S=\frac{2GM}{c^{2}}$$

is the Schwarzchild radius. Here, the denominator in (8) is equivalent to (7) above. Ref. [4] shows that we can define a *relativistic radius* of a mass sphere

(11) $$\bar{R}=\int_{0}^{R}\frac{dr}{\sqrt{1-\alpha^{2}r^{2}}}=\frac{1}{\alpha }\arcsin\left(\alpha R\right)$$

and a *relativistic volume* $\bar{V}$

(12) $$\bar{V}=\int_{0}^{R}\frac{r^{2}dr}{\sqrt{1-\alpha^{2}r^{2}}}\int_{0}^{\pi }\left(\int_{0}^{2\pi }\sin\theta d\psi \right)d\theta$$

The ranges of variables are R>S and r∈[0,R]

Ref. [4] shows that $\bar{V}>V$, and hence, for the same mass, the bulk relativistic density of a star $M/\bar{V}=\rho_{r}$ is less than its proper density $M/V=\bar{\rho }$. Of course, there are only two ways (notwithstanding conversions of mass to energy) density can change, and that is the flow of mass from one fixed volume into the next or a change in volume alone. In this work, we consider the latter of these mechanisms.

## 2. Equations for Mass and Moment of Inertia

The problem posed in this study is to determine the density distribution within a spherically symmetric neutron star, given only the following two constraints: the volumetrically averaged density $\bar{\rho }$ and the moment of inertia factor *f*. Note

(13) $$J=f{MR}^{2}$$

Here, *f* is a dimensionless factor proportional to the ratio of the actual moment of inertia of, for example, a celestial object *J* to the moment of inertia of a homogeneous sphere with the same mass and radius, respectively. Note also that $f<\frac{2}{5}$, and for the earth, f=0.3313.

Here, in order to deal with extremely dense objects, we recognize that space within these objects can be heavily curved, and thus, lengths and so on have to be adjusted for curvature. To do this, the metric (8) will be used, which avoids non-linearities later in the formulation. We believe this is justified based on (a) the level of spatial distortion, which is relatively small, about a 10% increase in a relative over a Euclidean volume for the objects studied; (b) the lack of closed-form solutions to GRT for inhomogeneous spherical objects; (c) the general lack of observational data for neutron stars (estimated radius, mass, and inertia); and (d) most importantly, the fact that curvature is determined by a large central mass so that fluctuations about this central mass to curvature should be negligible.

### 2.1. Constraint 1: Total Mass

Correcting for curvature (see, (12)), the total mass can be written in terms of the density as:

(14) $$M=4\pi \int_{0}^{R}\frac{\rho \left(r\right)r^{2}dr}{\sqrt{1-\alpha^{2}r^{2}}}$$

However, in the spherical body, ρ(r) is not given and is, in fact, the model to be determined. A standard approach in inverse (and numerical methods) is to divide the model ρ(r) into a series of discrete steps with a constant density imposed across each step. The resulting terms concerning density can then be taken outside the integral, and the integration is performed across each step. If the steps are considered small enough, the resulting sum of the integrals will converge. As [5] correctly points out, density can only have values from a discrete set. Therefore, when a sphere is subdivided into constant volume shells, we seek effective densities within these shells. The shells can be made arbitrarily small so as to be viewed as nearly continuous.

So, first, the spherical body is subdivided into *N* nearly equi-voluminous shells of density $\rho_{n}$, n=1,…,N. In the following developments, mass is conserved in that the sum of all masses contained in each of a series of shells equals the total mass of the system. What changes in each cell is the volume from ($\Delta V\to \Delta \bar{V}$) because of curvature, which, therefore, affects the density of matter in each shell. Using (14) leads to a total mass:

(15) $$M=4\pi \sum_{n=1}^{N}\rho_{n}\int_{r_{n-1}}^{r_{n}}\frac{r^{2}dr}{\sqrt{1-\alpha^{2}r^{2}}}$$

Integrating (15) leads to:

(16) $$M=4\pi \sum_{n=1}^{N}\rho_{n}\left[H\left(r_{n}\right)-H\left(r_{n-1}\right)\right]$$

where H(r) is [4]:

(17) $$H\left(r\right)=\frac{1}{2\alpha^{3}}\arcsin\left(\alpha r\right)-\frac{r}{2\alpha^{2}}\sqrt{1-\alpha^{2}r^{2}}$$

Without a loss of generality, dividing M in (16) by the volume of a sphere of coordinate radius R yields a volumetrically averaged (bulk) density

(18) $$\bar{\rho }=\frac{M}{\frac{4}{3}\pi R^{3}}=\frac{3}{R^{3}}\sum_{n=1}^{N}\rho_{n}\left[H\left(r_{n}\right)-H\left(r_{n-1}\right)\right]$$

and taking expected values yields

(19) $$\bar{\rho }=\frac{3}{R^{3}}\sum_{n=1}^{N}{\bar{\rho }}_{n}\left[H\left(r_{n}\right)-H\left(r_{n-1}\right)\right]$$

### 2.2. Constraint 2: Moment of Inertia Factor

For a second constraint, the moment of inertia *J* in radial coordinates is

(20) $$J=\int_{0}^{R}\frac{r^{2}dr}{\sqrt{1-\alpha^{2}r^{2}}}\int_{0}^{\pi }\sin\theta d\theta \int_{0}^{2\pi }\rho \left(r,\theta ,\psi \right)\delta {\left(r,\theta ,\psi \right)}^{2}d\psi$$

For radial dependences only and correcting for relativistic effects:

(21) $$J=\frac{8\pi }{3}\int_{0}^{R}\frac{\rho \left(r\right)r^{4}dr}{\sqrt{1-\alpha^{2}r^{2}}}$$

Discretizing ρ as before yields

(22) $$J=\frac{8\pi }{3}\sum_{n=1}^{N}\rho_{n}\int_{r_{n-1}}^{r_{n}}\frac{r^{4}dr}{\sqrt{1-\alpha^{2}r^{2}}}$$

and, finally, to:

(23) $$J=\frac{8\pi }{3}\sum_{n=1}^{N}\rho_{n}\left[P\left(r_{n}\right)-P\left(r_{n-1}\right)\right]$$

Here, P(r) is

(24) $$-\frac{\sqrt{1-\alpha^{2}r^{2}}\left(2\alpha^{3}r^{3}+3\alpha r\right)-3\arcsin\left(\alpha r\right)}{\alpha^{5}}$$

Equation (24) can be shown to be a primitive of the integrand in (22). Equating (13) to (23), dividing by volume to yield densities, and taking expected values yields (see (18) and (19)):

(25) $$\frac{2}{R^{5}}\sum_{n=1}^{N}{\bar{\rho }}_{n}\left[P\left(r_{n}\right)-P\left(r_{n-1}\right)\right]=f\bar{\rho }$$

Notice that the data are completely inadequate with respect to the mathematical problem of determining $\rho_{n}$ the model parameters from only two constraints. In the subsequent development below, the vector $m=(\rho_{1},\rho_{2},\ldots ,\rho_{N})$.

## 3. Minimum Relative Entropy (MRE) Solution

The precursor methodology to that discussed in this paper was first detailed by [5] as an example application of maximum entropy (MaxEnt: [6,7] to the density profile of the Earth (see also [8,9]). Ref. [10] also used MaxEnt for a series of planetary problems and presented some pros and cons of the method. Alternatively, in this work, we use a minimum relative entropy formulation (MRE; [11,12]). MRE has the advantage over MaxEnt in that it allows for non-positive models, hard bounds (if available), and a prior bias in terms of a prior pdf of the model. Ref. [13] also solved for the density of the Earth with MRE and compared those solutions to MaxEnt and Bayesian methods.

Given information about a star in terms of the total amount of mass and the moment of inertia, what is the most probable distribution of matter within the star? Specifically, given a prior pdf of a model, p(m), subject to constraints, we can obtain a posterior estimate of the model m by computing the expected values [14]. In the present application, we want to find the pdf q(m), which has minimum relative entropy with respect to the prior. The constraints are of the form:

(26) $$\int q^{†}\left(m\right)g_{j}\left(m\right)dm={\bar{d}}_{j}$$

where $g_{j}\left(m\right)$ are known kernel functions and ${\bar{d}}_{j}$, j=1…M are known data in the form of moments. The statistical problem posed here is to determine an estimate of the pdf $q^{†}\left(m\right)$, q(m) based on the information provided ([11]).

Consider the discrete form of the linear inverse problem (26), where **d** represents a discrete set of known data, g is a matrix of the known kernel functions, and m is the vector of unknown “true” model parameters. It is our goal to obtain an estimate $\hat{m}$ of m that satisfies the constraints and is distinguishable from all other $\hat{m}$ by a rational criterion. Also, any a priori estimate s of m should be included in the inversion scheme. The discretization can be written as

(27) $$d_{j}=\sum_{n=1}^{N}g_{jn}m_{n}$$

And taking expected values yields

(28) $${\bar{d}}_{j}=\int_{M}q\left(m\right)\left[\sum_{n=1}^{N}g_{jn}m_{n}\right]dm$$

where q(m) is the posterior pdf of m, and the integration is over all the allowed values of m. At this point, the values of m are allowed in the range of (L,U), where *U* is an upper bound to the model, and consequently, $\int_{M}$ implies $\int_{L}^{U}$. Equation (28) is now of the required form of (26). Refs. [12] and [15] determined the specific form of q(m). We now denote $\hat{m}$ as the expected value of the random vector m. That is, $\hat{m}=\int_{M}mq\left(m\right)dm$.

Ref. [14] show that the mean values are functions of a number of Lagrange multipliers (λ; one for each constraint, j=1,M), so that

(29) $${\bar{d}}_{j}=\sum_{n=1}^{N}g_{jn}{\hat{m}}_{n}\left(\lambda \right)$$

and the (λ) values are subsequently found by a Newton–Raphson scheme (e.g., [16]). For a complete development, see [13].

The reader should also note that the probabilities obtained are conditional on the imposed moments, namely, M and *f*, and also the radius R. It must be emphasized these data are considered complete and accurate.

### Prior Probability Assignment

Before one can proceed further, we need to establish an appropriate prior probability p(m) for the model parameters, $\rho_{n}$. Ref. [15] thoroughly discuss the estimation of prior probabilities that are appropriate in many physical settings. It is acknowledged that astronomic databases are such that reasonable upper and lower bounds on parameters can be made in many cases. If only these limits are known, then this implies a boxcar pdf (uniform distribution between an upper and lower bound). Suppose additional information, such as an informed estimate, limited tests, or simple back calculation (calibration), becomes available, which we will call s. As shown by [15], this implies a pdf that is a multivariate, uncorrelated truncated exponential p(m) with expected value s. This pdf basically has an upper and lower bound and a mean value for each parameter. In this manner, the prior pdf has the most freedom in assigning realizations of the process and is the most uncommitted with respect to unknown information. Ref. [17] emphasized that the choice of prior models with expected values s allows us to explore the limits of model space.

## 4. Numerical Experiments

The examples presented herein are not carried out in an exhaustive manner. They were selected on their basis as interesting subjects and ones for which some data are available and appropriate for inclusion in this technical note. The particular significance of prior information, in terms of an upper and lower bound (hard constraints) and soft constraints (prior mean values), allows us to explore model space efficiently while fitting the observations.

In a numerical experiment, we will use the data from star PSR J0737-3039A, which has a mass of $2.6\times 10^{30}$ kg and a radius of 13.75 km [18]. This star has a bulk density of $2.4\times 10^{14}$ gm/cc. If nothing else is known (see [15]), a value for s the vector of prior means is set at a uniform value in the radial direction, between an upper and lower bound. This will be referred to as a “flat” prior.

Next, we need upper and lower bounds on density. Ref. [19] note that supra-nuclear density determinations at the core of a neutron star are not possible with current laboratory experiments, and the equation of state is poorly constrained past $2.8\times 10^{14}$ gm/cc. Ref. [20] suggests a value of $1.4\times 10^{15}$ gm/cc, an assumed value for quark–gluon plasma that is considered to be present at the core of such a star (see also Figure 1). We will, however, set the lower bound to be 0 gm/cc and the upper bound at $1.0\times 10^{16}$ gm/cc, and it is recognized that further astrophysical observations are going to be essential for resolving these issues [19].

The MOI factor *f* is taken as 0.258–0.324 based on the work of [18] and more recently [21], who list a range of *J* = 1.30–$1.63\times 10^{45}$ g cm^2^ for the star PSR J0737-3039A. Results from [19] list a value of the MOI at $1.15\times 10^{45}$ g cm^2^, which is based on the existence of a universal relation for neutron stars and an analogy to GW170817. A less restrictive bound from the same analysis corresponds to $1.67\times 10^{45}$ g.cm^2^, similar to [18] upper bound. In the following analysis, we will use the range 1.30–$1.63\times 10^{45}$ g cm^2^.

**Figure 2:**
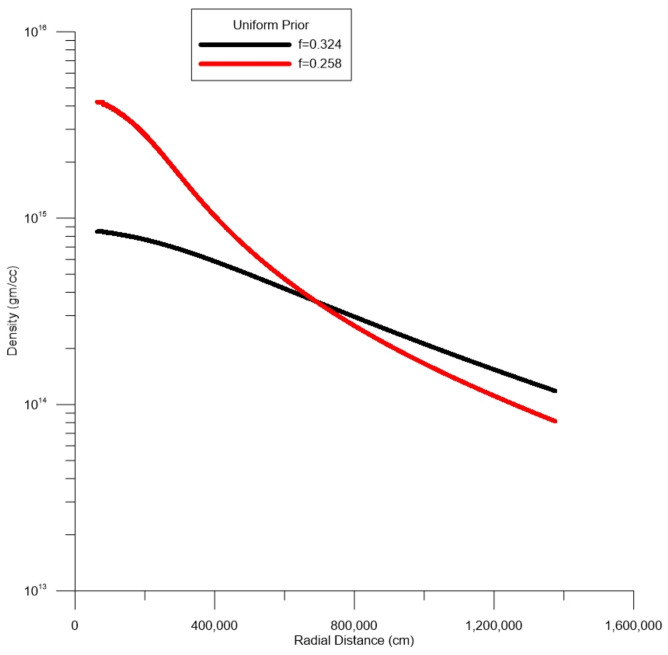
Star J0737-3039A. Expected values of density for different values of moment of inertia (MOI) factors, *f*. The dashed line is the expected value of prior probability, which is a uniform prior $2.4\times 10^{14}$ gm/cc in the radial direction.

In the first series of simulations, the results are shown in Figure 2 for f=0.324 and a flat prior of $2.4\times 10^{14}$ gm/cc, which is within nuclear density values. Here, 10,000 unknowns are sought and the output density ranges from about $1\times 10^{14}$ to $8\times 10^{14}$ gm/cc. Interestingly, at no time do densities cross over $1\times 10^{15}$ gm/cc. For the most part, densities >$6\times 10^{14}$ gm/cc are shown at radial dimensions of less than about 4 km. Figure 2 also shows results when f=0.258, and under the same assumptions as the previous case (flat prior), densities closer to the core are pushed higher, as one might expect and peak at slightly over $4\times 10^{15}$ gm/cc.

**Figure 3:**
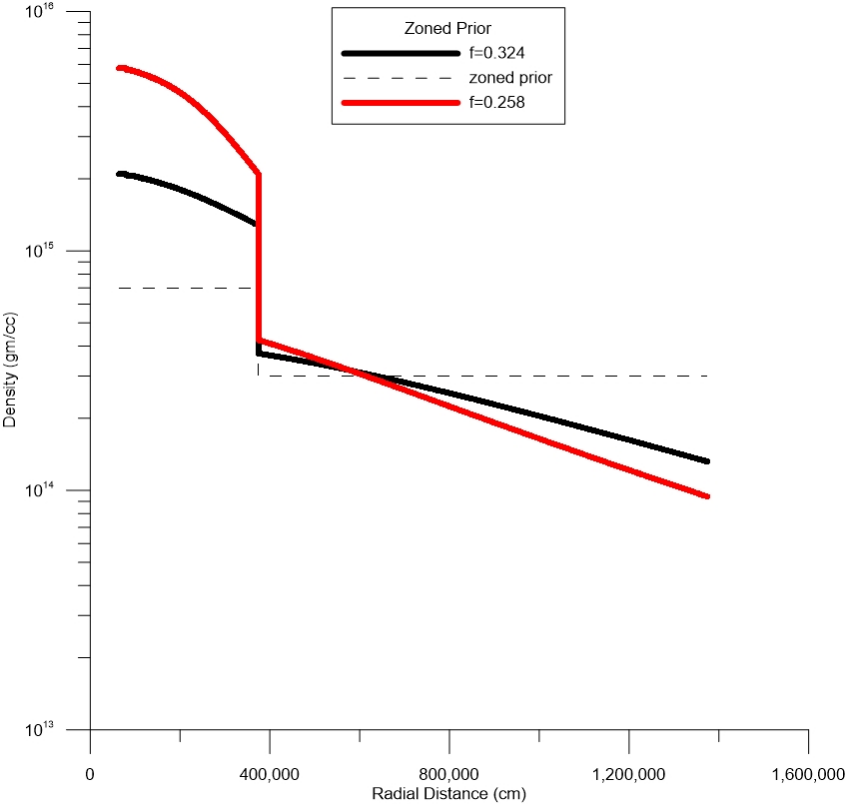
SR J0737-3039A. Expected values of density for two different values of moment of inertia factor *f*. The dashed line is the expected value of prior probability, which, in this case, is a zoned conceptual model loosely based on the conceptual model of Figure 1.

The MRE approach allows us to consider prior values based on other studies or other kinds of “soft” information. Figure 1 suggests that there may exist various zones, such as inner and outer cores, perhaps with constant densities therein. We can introduce a zoned model loosely based on a generic picture of a neutron star (Figure 1 and Figure 3, shown as a dashed line). The prior mean value is defined as between 0 and 3.75 km: $7.0\times 10^{14}$ gm/cc; 3.75 and 13.75 km: $3.0\times 10^{14}$ gm/cc; and >13.75 km: $4.0\times 10^{11}$ gm/cc. Note densities at the core are pushed even higher to about $4\times 10^{15}$ gm/cc for f=0.258. Figure 3 also shows the results when the same zone model is used with a higher value of f=0.324. Internal densities are pushed into nuclear values only at the core region and a sudden jump is noted at the postulated inner core boundary. These simulations show a qualitative equivalence to that of the conceptual model of Figure 1. In both cases, for different values of *f*, the zoned picture of Figure 3 produces lower relative entropies than those of the flat prior solutions of Figure 2.

**Figure 4:**
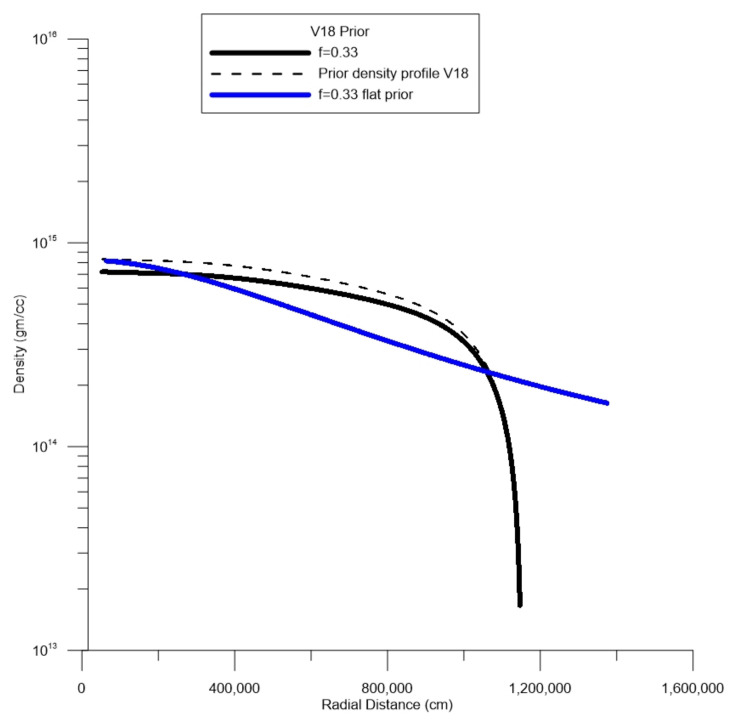
Assumed conceptual model of a neutron star studied by [20]. The expected value of density for assumed values of moment of inertia factor *f*. The dashed line is the expected value of prior probability from nuclear model V18. The dashed line is a uniform prior in the radial direction.

In Figure 4, a hypothetical case is shown [20] which is from a neutron star of 1.4 solar masses, with an estimated radius of 11.5 km. We show a density profile based on a flat prior and another prior based on one of the microscopic nuclear models that [20] investigated: V18. In these simulations, one can see the dashed line, which is the prior, the solid black line, and the posterior mean value. In this simulation, a value of f=0.33 was assumed based on that of the Earth and the upper bound was set to $1\times 10^{16}$ gm/cc. In this figure, no attempt at any calibration was taken. This simulation is insightful and shows a reasonable equivalence to that of the models of [20]. If true, this would imply a moment of inertia close to $1.2\times 10^{45}$ g cm^2^ for this hypothetical case.

## 5. Discussion and Limitations

As mentioned, Ref. [4] concluded that gravity cannot collapse objects in neutron stars higher than nuclear densities. For an upper limit, that might be $1.4\times 10^{15}$ gm/cc. The simulations show that the values of ρ are pushed higher than this value for lower ranges of *f*. This would suggest that the values of the moment of inertia for PSR J0737-3039A might be closer to $1.63\times 10^{45}$ g.cm^2^ in order for lower core densities to be true. If recent values of MOI by [19] are more appropriate at $1.15\times 10^{45}$ g.cm^2^, this then suggests core densities greater than $4\times 10^{15}$ gm/cc.

It was noted that the probabilities and the mean values of density obtained are based on strict equality to the imposed moments (28), namely, M and *f*, and also the radius R. The results presented herein show great sensitivity to the imposed values of MOI, particularly in the core regions. As [19] note, future astrophysical observations are essential for resolving values of the MOI and their ability to constrain neutron star EOS values.

This work has a number of limitations. It is assumed here we are dealing with a spherically symmetric stellar object comprising *N* equi-volume thin shells. Of course, this is an ideal conceptual model. The adjustments in the preceding equations that allow for curvature dependencies rely on solutions of the Schwarzchild metric, which, in itself, is derived on the basis of a static spacetime, a homogeneous, incompressible fluid. [22] argues that local volume elements depend on curvature, and thus, density may change with the metric without any flow of mass. In this work, it can be shown that not all of the density differences can be explained by this mechanism.

## 6. Conclusions

It should be clear we are seeking the expected value of a series of effective densities in a thin-shelled, spherically symmetric neutron star. Recall that the expected value is the best estimate of a random variable’s behavior in a mean-square sense. Is this density model of a symmetric sphere applicable to a star such as PSR J0737-3039A? The solutions appear to be generally consistent with neutron star models examined in this work. This is certainly not the final answer; rather, it is an exploratory–numerical experiment to show how minimum relative entropy (MRE) can be used to extract information about a model with limited constraints. These density models lead to quantitative statements about qualitative interpretations, and as time goes on, any internal density models must satisfy the two constraints posed. For example, any postulated density profile of a neutron star should, through (14), satisfy an external observation of total bulk density M divided by the volume of a sphere of radius R. Also, since the model presented here is probabilistic, it can be established that density at a certain depth is constrained within a certain confidence limit. We note that the MRE method can explore model space over the whole range of entropies allowed by the data constraints. This research points to the need for more comparisons and analysis, such as direct comparisons with TOV-based solutions, and understanding the role of different EOS models.

Note the following important observations made by [5] when discussing the related MaxEnt procedure, which are appropriate when considering MRE: “The Maxent concept is, of course far from being exhaustively in this text. In fact many lines of thought have only been sketched, many problems have only been touched and require more detailed analysis. It is hoped that this paper provokes further investigation of the Maxent concept of handling inverse problems. In any case it should be clear that this method must not be used indiscriminately and in no case substitute, but only complement observations, measurements and physical reasoning.”

## Data Availability

All of the data used herein are from publicly available datasets or published sources. See SIMBAD (Set of Identifications, Measurements and Bibliography for Astronomical Data) for PSR J0737-3039A and also Miao et al. (2022) [21]. See Sammarruca (2014) [20] for data on other stars.
